# Genome-wide association study identifies four SNPs associated with response to platinum-based neoadjuvant chemotherapy for cervical cancer

**DOI:** 10.1038/srep41103

**Published:** 2017-01-25

**Authors:** Xiong Li, Kecheng Huang, Qinghua Zhang, Jin Zhou, Haiying Sun, Fangxu Tang, Hang Zhou, Ting Hu, Shaoshuai Wang, Yao Jia, Ru Yang, Yile Chen, Xiaodong Cheng, Weiguo Lv, Li Wu, Hui Xing, Lin Wang, Shasha Zhou, Yuan Yao, Xiaoli Wang, Quzhen Suolang, Jian Shen, Ling Xi, Junbo Hu, Hui Wang, Gang Chen, Qinglei Gao, Xing Xie, Shixuan Wang, Shuang Li, Ding Ma

**Affiliations:** 1 Department of Obstetrics and Gynecology, Tongji Hospital, Tongji Medical College, Huazhong University of Science and Technology, Wuhan, P.R. China; 2 Department of Gynecology & Obstetrics, the Central Hospital of Wuhan, Tongji Medical College, Huazhong University of Science and Technology, Wuhan, P.R. China; 3 Cancer Center, Renmin Hospital of Wuhan University, Wuhan, Hubei, P.R. China; 4 Department of Gynecologic Oncology, Hunan Province Tumor Hospital, Changsha, P.R. China; 5 Women’s Reproductive Health Laboratory of Zhejiang Province, Zhejiang, P.R. China; 6 Department of Obstetrics and Gynecology, Xiangfan Central Hospital, Tongji Medical College, Huazhong University of Science and Technology, Xiangfan, Hubei, P.R. China

## Abstract

To identify genomic markers associated with the response to neoadjuvant chemotherapy (NACT) in patients with cervical cancer, we performed a three-stage genome-wide association study (GWAS) in the Han Chinese population. A total of 596 patients with stage IA2-IIIB cervical cancer were enrolled in this study. One single nucleotide polymorphism (SNP) (rs6812281, per allele OR = 2.37, *P* = 9.0 × 10^−9^) located at 4q34.3 reached GWAS significance (*P* < 5.0 × 10^−8^). Another three SNPs, rs4590782 (10q26.2, *P* = 1.59 × 10^−5^, per allele OR = 0.48), rs1742101 (14q32.11, *P* = 7.11 × 10^−6^, per allele OR = 0.52), and rs1364121 (16q23.3, *P* = 3.15 × 10^−6^, per allele OR = 1.98), exhibited strong evidence of associations with response to neoadjuvant chemotherapy. Patients with a C allele (CT + CC) of rs4590782 had better 5-year overall survival rates (82.9% vs. 75.8%, *P* = 0.083) and 5-year disease-free survival rate (80.8% vs. 72.7%, *P* = 0.021) than those without a C allele. Our findings help to characterize the genetic etiology of the response to neoadjuvant chemotherapy in patients with cervical cancer.

Cervical cancer is the second most commonly diagnosed cancer and third leading cause of cancer death in females in less developed countries^1^. Neoadjuvant chemotherapy (NACT) was proven to be an effective treatment for cervical cancer^2^,^3^. However, approximately 20% of patients do not respond to NACT. The ability to predict the NACT non-responders could save time and allow selection of more suitable treatments.

In previous studies, no clinical factors have shown a strong correlation with the response to NACT^2^,^3^. Additionally, there has been no bio-marker proven to effectively predict the response to NACT. On one hand, using the candidate method has been difficult due to our limited understanding of the underlying biology; on the other hand, neoadjuvant chemotherapy regimens in different studies have been too inconsistent to obtain similar conclusions. Despite these difficulties, squamous cell carcinoma antigen (SCC-Ag) has been proven to be related to the response to NACT in several reports^4^,^5^,^6^. Hyun Hoon Chung *et al*.^7^ have reported that a single nucleotide polymorphism (SNP) at codon 399 of the XRCC1 gene influences the response to platinum-based NACT in patients with cervical cancer and large tumors. Similar results have been reported by Xiao-dong Cheng *et al*.^8^,^9^. However, the sample size in these studies was limited, and the predictive power was insufficient to guide clinical practice.

In this report, we conducted a genome-wide association study (GWAS) of cervical cancer in the Han Chinese population to discover genetic variants associated with differential responses to platinum-based NACT.

## Results

### Characteristics of the patients

The clinical characteristics of the 596 patients are shown in Table 1. A total of 451 (75.7%) patients were evaluated as responsive to NACT, and 145 (24.3%) patients were evaluated as non-responsive to NACT. Patients were divided into three sets: the discovery, follow-up 1, and follow-up 2 sets with response rates of 70.8%, 74.9%, and 85.2%, respectively. Most of the patients (523, 87.8%) were diagnosed as FIGO stage IB2 (124, 20.8%), IIA (146, 24.5%), or IIB (253, 42.4%). The mean age of all the patients was 46.16 years old with a range of 23 to 72 years old. A total of 553 (92.8%) patients were diagnosed with squamous cell carcinoma. Only 43 (7.2%) of the 596 patients were diagnosed with adenocarcinoma or adenosquamous carcinoma. When all 596 patients were analyzed, the response to NACT was associated with tumor size (*P* < 0.001) but was not associated with age (*P* = 0.40), FIGO stage (*P* = 0.22), or histology (*P* = 0.59).

### Genetic association analysis of the response to neoadjuvant chemotherapy in the discovery stage

**Figure 1:**
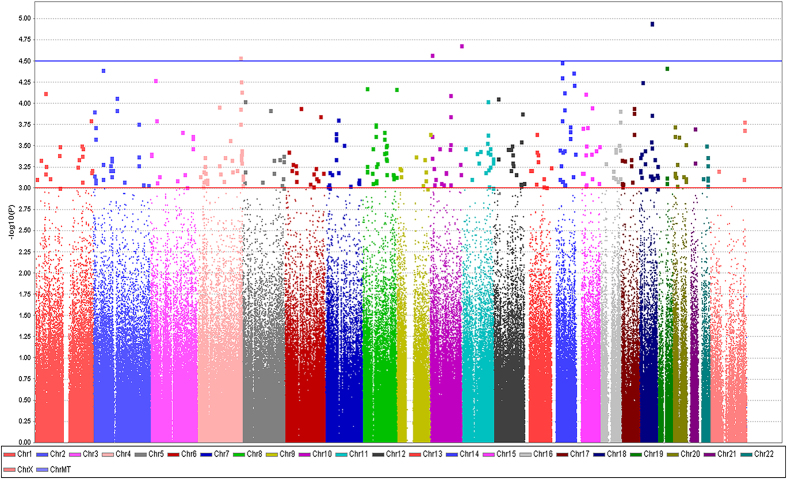
Manhattan plot showing the association between the scatter plot of P values in −log10 scale and the response to neoadjuvant chemotherapy in the discovery set.

A total of 226 patients, including 160 responders and 66 non-responders, from three hospitals were enrolled in the discovery stage (Table S1). The top 100 ranked SNPs associated with response to neoadjuvant chemotherapy in the discovery stage are shown in Table S2. A total of 23 SNPs were selected from the top 100 SNPs as candidates to be validated in the follow-up 1 stage. All 23 SNPs had *P* values ≤ 3.0 × 10^−4^ (Table S3). A Manhattan plot (Fig. 1) shows a graphical summary of the genome-wide association results. We analyzed the quality of our data via a quantile-quantile plot (Figure S1) and used principal component analysis to reveal any population stratification (Figure S2).

### Genetic association analysis of the response to NACT in the follow-up 1 stage

We selected 235 patients (176 responders and 59 non-responders) from Tongji Hospital, Xiangyang Central Hospital, and the Women’s Reproductive Health Laboratory of Zhejiang Province to perform the follow-up 1 replicate (Table S1). Detailed information for the follow-up 1 study is shown in Table S4. Five SNPs: rs6812281 in 4q34.3, rs4590782 in 10q26.2, rs8019419 in 14q22.1, rs1742101 in 14q32.11, and rs1364121 in 16q23.3 were associated with the response to NACT (*P* < 0.05). The other 18 SNPs were not associated with the response to NACT (*P* > 0.05). When combining data from the discovery and follow-up 1 stages, four (rs6812281, rs4590782, rs1742101, rs1364121) of the five SNPs had *P* values < 5.0 × 10^−5^ (Table S5).

### Genetic association analysis of the response to NACT in the follow-up 2 stage

A total of 135 patients (115 responders and 20 non-responders) from Tongji Hospital were used to perform the follow-up 2 replicate. The four SNPs (rs6812281, rs4590782, rs1742101, and rs1364121) with *P* values < 0.05 in follow-up 1 were genotyped in follow-up 2. rs6812281 on chromosome 4 and rs1364121 on chromosome 16 showed association with the response to NACT (*P* < 0.05, Table S6).

### Genetic association analysis combining all three stages

We combined the discovery, follow-up 1, and follow-up 2 stages to analyze the association between the four SNPs and the response to NACT. rs6812281 (*P* = 9.00 × 10^−9^) showed a genome-wide level (*P* < 5.0 × 10^−8^) of association with the response to neoadjuvant chemotherapy. The other three SNPs: rs4590782 (*P* = 1.59 × 10^−5^), rs1742101 (*P* = 7.11 × 10^−6^), and rs1364121 (*P* = 3.15 × 10^−6^) showed relatively strong correlation with the response to neoadjuvant chemotherapy (Table S7, Table 2). Despite reaching genome-wide significance, rs6812281 in 4q34.3 is located in an intergenic area with no gene within ±500 kb. rs4590782 in 10q26.2 is located in an intergenic region with genes BUB1P1, CLRN3, PTPRE, MKI67, LINC01163, DOCK1, NPS, and FOXI2 within ±500 kb. rs1364121 in 14q32.11 is in an intronic region of the TTC7B gene with genes CALM1, NRDE2, PSMC1, RPS18P2, RPS18P2, RPS6KA5, RNU7-30P, C14orf159, and SNORA11B nearby (Table 2). rs1364121 in 16q23.3 located in an intronic region of the CDH13 gene with genes MIR3182, HSBP1, MLYCO, OSGIN1, NECAB2, SLC38A8, RNA5SP432, MBTPS1, HSDL1, DNAAF1, TAF1C, and ADAD2 nearby.

**Figure 2:**
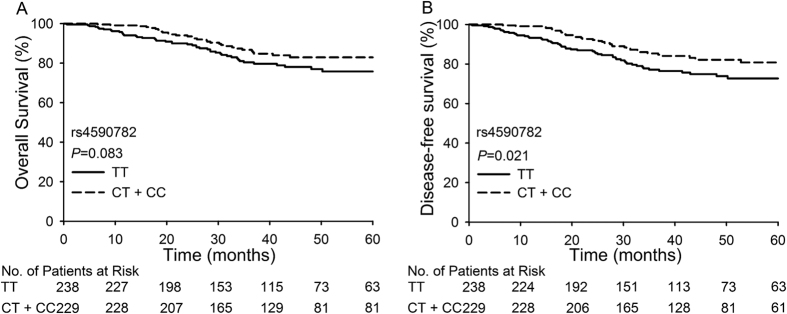
Overall survival and disease-free survival of patients with different genotypes of rs4590782. (**A**) Comparison of overall survival between patients with the TT genotype and patients with CT or CC genotypes; (**B**) Comparison of disease-free survival between patients with the TT genotype and patients with CT or CC genotypes.

None of the four SNPs was associated with the FIGO stage, histology type, or tumor size (Table S8). When we performed a multivariate logistic analysis adjusting for tumor size, three of the four SNPs (rs6812281, rs4590782, rs1364121) and tumor size proved to be independent predictive factors of the response to neoadjuvant chemotherapy. rs6812281 with a T allele (GT + TT, P = 2.47 × 10^−6^, OR = 2.621), rs1364121 with an A allele (GA + AA, P = 1.55 × 10^−3^, OR = 1.937), and tumor size ≥4 cm were related to a poor response to NACT. rs4590782 with a C allele (CT + CC, P = 3.04 × 10^−4^, OR = 0.472) was significantly associated with a good response to NACT. We also analyzed the relationship between these four SNPs and the survival of patients. Patients with a C allele (CT + CC) of rs4590782 had better 5-year overall survival (82.9% vs. 75.8%, *P* = 0.083) and 5-year disease-free survival (80.8% vs. 72.7%, *P* = 0.021) rates than those with the TT variant of rs4590782 (Fig. 2).

## Discussion

In this study, we used a three-stage analysis, including a total of 596 cervical cancer patients, to identify genome-wide associations for the response to neoadjuvant chemotherapy. We identified one SNP (rs6812281) on 4q34.3 that reached genome-wide levels of statistical significance (*P* = 9.00 × 10^−9^). Another three SNPs (rs1364121 on 16q23.3, rs1742101 on 14q32.11, and rs4590782 on 10q26.2) showed weaker associations that did not reach genome-wide levels of statistical significance.

In previous studies, genetic polymorphisms have been shown to be associated with the response to platinum-based chemotherapy in gynecological cancers^10^. Cisplatin is an agent used to treat several types of cancers. Cisplatin causes DNA lesions via the formation of intrastrand and interstrand crosslinks, resulting in the activation of various signal-transduction pathways that block cellular processes, such as replication and transcription^11^,^12^,^13^. Genetic changes that modify the cellular phenotype could explain some of the variability in the response and toxicity of cisplatin chemotherapy^11^,^14^,^15^. Genetic polymorphisms, including XRCC1 194A > T, XRCC1 R399Q, GGH 401C > T, were reported to be associated with response to platinum-based NACT in patients with cervical cancer^7^,^8^,^9^. In this genome-wide association study, we also found several SNPs associated with this response.

For the most significant SNP in 4q34.3, rs6812281, there is no gene within ±500 kb. It is difficult to evaluate how rs6812281 might impact the response to NACT. The SNP rs4590782 in 10q26.2 is also located in an intergenic area. It was associated with both the response to NACT and survival. Patients with a C allele of rs4590782 seem to have a better 5-year overall survival and 5-year disease-free survival than patients with the TT variant. MKI67 is one of the genes near rs4590782 and has been studied extensively in breast cancer. Ki-67 is a nuclear protein forming part of the DNA replicase complex^16^,^17^,^18^ and is widely used to detect the proliferation activity of tumors^19^. In some previous studies, the expression of Ki-67 before treatment has been associated with the response to NACT in patients with cervical cancer^20^,^21^,^22^. rs4590782 may have some relationship with Ki-67. This interaction needs to be studied.

TTC7B is a member of the tetratricopeptide repeat (TPR) gene family. Tetratricopeptide repeats consist of tandem arrays of highly degenerate 34-amino acid repeats that are predicted to form extended superhelical arrangements^23^. These TPR domains function as protein–protein interaction modules for macromolecular complexes involved in numerous cellular processes, including transcriptional regulation, mRNA processing, protein folding, and translocation^24^. However, to date, there are no reports on the function of TTC7B.

CDH13 is a special cadherin cell adhesion molecule. Because they mediate adhesion between normal cells, cadherins play an important role in the establishment of cell polarity, by which they induce cell cycle arrest and inhibit tumor invasion and tumor amplification. The methylation level of RASSF1A and CDH13 promoter regions can reflect the drug sensitivity of tumors to individual treatments^25^. CDH13 hypermethylation is associated with increased risk and poor survival in nonsmall cell lung cancer (NSCLC)^26^. The hypermethylation of the CDH13 promoter is an early event in the initiation and progression of cervical neoplasia^27^. The genotype of rs1364121, which is located in an intron of CDH13, may influence the expression of CDH13.

Although we performed a genome-wide association analysis of response to neoadjuvant chemotherapy in patients with cervical cancer, our sample size was limited. The most significant SNP rs6812281 was located in a gene desert, and it is difficult to explain its association with the response to NACT. The other three SNPs (rs4590782, rs1742101, and rs1364121) showed some association with the response to NACT, but did not reach GWAS significance. Our study showed that there are genetic polymorphisms associated with the response to neoadjuvant chemotherapy in patients with cervical cancer. The interaction between the significant SNPs and their surrounding genes needs to be explored. Considerable further research is required to advance these findings, and these results should be validated by other researchers.

## Materials and Methods

### Study samples

We performed a three-stage study, and a total of 596 patients were enrolled. The discovery, follow-up 1, and follow-up 2 stages included 226 patients (160 responders and 66 non-responders), 235 patients (176 responders and 59 non-responders), and 135 patients (115 responders and 20 non-responders), respectively. The patients in this multi-center study were mainly from Tongji Hospital (Wuhan, China), Xiangyang Central Hospital (Xiangyang, China), and the Women’s Reproductive Health Laboratory of Zhejiang Province (Zhejiang, China) (Table S1) and were diagnosed between January 1, 2009 and May 29, 2014. Patients in the discovery and follow-up 1 stages were from Tongji Hospital, Xiangyang Central Hospital, and Zhejiang Province and were diagnosed between January1, 2009 and December 31, 2012. Patients in the follow-up 2 stage were from Tongji Hospital (Table S1) and were diagnosed between January1, 2013 and May 29, 2014. All of the enrolled patients meet the following inclusion criteria: (1) have stage IA-IIIB cervical cancer according to the Federation of Gynecology and Obstetrics (FIGO); (2) were aged from 18 to 75 years; (3) did not receive hysterectomy, pelvic radiotherapy or concurrent chemoradiotherapy before neoadjuvant chemotherapy; (4) received platinum-based neoadjuvant chemotherapy; (5) have detailed information on tumor size before and after neoadjuvant chemotherapy. This study was authorized and approved by the Ethics Committee of Tongji Hospital of Tongji Medical College, Huazhong University of Science and Technology, PR China. We confirm that all participants have provided their written informed consent in this study. All methods were carried out in accordance with relevant guidelines and regulations.

### Neoadjuvant chemotherapy regimens

Generally, all patients received 1–2 cycles of neoadjuvant chemotherapy every 28 days. However, the cycles of NACT that the patients received were based on the physician’s judgment. The regimens for NACT consisted of paclitaxel and cisplatin (TP) or irinotecan and cisplatin (CP). The response to neoadjuvant chemotherapy was evaluated by two experienced doctors according to the World Health Organization (WHO) criteria: complete response (CR), complete disappearance of the tumor; partial response (PR), a decrease in tumor volume of 50% or more; stable disease (SD), a less than 50% reduction in tumor volume; progressive disease (PD), an increase of 25% or more in volume or the appearance of new lesions (2).

### DNA extraction

Blood samples from all participants were preserved by EDTA disodium salts (EDTA-2Na). Genomic DNA was extracted from peripheral blood by standard procedures using the QuickGene DNA whole blood kit (Fujifilm) and the Flexi Gene DNA kit (Qiagen). The extracted blood DNA was diluted to concentrations of 50 ng/μl for genome-wide genotyping or 20 ng/μl for the validation studies.

### Genotyping and quality control

In the discovery stage, we used the Affymetrix Axiom™ Genome-Wide CHB1 Array for 657,178 single nucleotide polymorphisms (SNPs) to genotype 231 patients. After the principal component analysis (PCA) and standard quality control that removed SNPs with call rates <98% (61,323 SNPs), SNPs for which the minor allele frequency (MAF) was <1% (43,000 SNPs) or SNPs deviating significantly (P ≤ 1 × 10^−5^) from Hardy-Weinberg Equilibrium in controls (3,540 SNPs), a total of 554,524 SNPs were analyzed in 226 patients (160 responders and 66 non-responders). A total of 85 SNPs had *P* ≤ 3.0 × 10^−4^ in the discovery stage. We selected 23 SNPs with minimum *P* values, and ignored the SNPs with high linkage disequilibrium (LD; r^2^ ≥ 0.3) with the selected SNPs. In follow-up 1 and follow-up 2, the Sequenom MassARRAY system (Sequenom Inc) and TaqMan assays (Applied Biosystems) were used to perform genotyping according to the manufacturers’ instructions.

### Statistical analysis

Either Pearson’s chi-square test or Fisher’s exact test was used to assess the relationship between the clinical characteristics and the response to NACT. The comparison of overall survival (OS) and disease-free survival (DFS) curves of patients with different genotypes were performed using the Kaplan-Meier method with the log-rank test. A *P* value of less than 0.05 was considered to be significant. EIGENSTRAT was used to perform the principal component analysis (PCA) of population stratification. The inflationary effect of population stratification on the GWAS results (λ = 1.004 and λ_1000_ = 1.044, Figure S1) was calculated by PLINK. SNPs associated with response to NACT were analyzed using logistic regression analysis. The SNPs with *P*-values ≤ 3.0 × 10^−4^ in the discovery stage were selected to be validated in follow-up 1. In follow-up 1, SNPs with *P*-values < 0.05 were considered to be significant. When combining the discovery, follow-up 1, and follow-up 2 stages, *P* < 5.0 × 10^−8^ was used as the genome-wide significance threshold. A quantile-quantile plot was created to evaluate the overall significance of the GWAS results. All statistical analyses were performed using SPSS software 13.0 (Chicago, IL, USA), PLINK Version 1.07, and R.

## Additional Information

**How to cite this article**: Li, X. *et al*. Genome-wide association study identifies four SNPs associated with response to platinum-based neoadjuvant chemotherapy for cervical cancer. *Sci. Rep.* **7**, 41103; doi: 10.1038/srep41103 (2017).

**Publisher's note:** Springer Nature remains neutral with regard to jurisdictional claims in published maps and institutional affiliations.

## Supplementary Material

Supplementary Information

## Figures and Tables

**Table 1:** Clinical characteristics of patients in different stages.

Characteristics	Total				Discovery set			
	Total	Responders	Non-responders	*P*	Total	Responders	Non-responders	*P*
No.	596	451(75.7%)	145(24.3%)		226	160(70.8%)	66(29.2%)	
Age, year	46.16 (23–72)	46.01(23–69)	46.64(23–72)	0.40	46.33(23–72)	45.88(25–69)	47.42(23–72)	0.18
FIGO stage								
IA2	2(0.3%)	1(0.2%)	1(0.7%)		0(0.0%)	0(0.0%)	0(0.0%)	
IB1	30(5.0%)	25(5.5%)	5(3.4%)		11(4.9%)	9(5.6%)	2(3.0%)	
IB2	124(20.8%)	92(20.4%)	32(22.1%)		53(23.5%)	32(20.0%)	21(31.8%)	
IIA	146(24.5%)	117(25.9%)	29(20.0%)		67(29.6%)	47(29.4%)	20(30.3%)	
IIB	253(42.4%)	192(42.6%)	61(42.1%)		85(37.6%)	64(40.0%)	21(31.8%)	
IIIA	13(2.2%)	9(2.0%)	4(2.8%)		5(2.2%)	5(3.1%)	0(0.0%)	
IIIB	28(4.7%)	15(3.3%)	13(9.0%)		5(2.2%)	3(1.9%)	2(3.0%)	
IA2-IIA	302(50.7%)	235(52.1%)	67(46.2%)	0.22	131(58.0%)	88(55.0%)	43(76.8%)	0.16
IIB-IIIB	294(49.3%)	216(47.9%)	78(53.8%)		95(42.0%)	72(45.0%)	23(23.2%)	
Histology								
Squamous cell carcinoma	553(92.8%)	417(92.5%)	136(93.8%)	0.59	210(92.9%)	148(92.5%)	62(93.9%)	0.99
Adenocarcinoma^a^	43(7.2%)	34(7.5%)	9(6.2%)		16(7.1%)	12(7.5%)	4(6.1%)	
Tumor size								
<4	174(29.2%)	149(33.0%)	25(17.2%)	< 0.001	53(23.5%)	47(29.4%)	6(9.1%)	< 0.001
≥4	422(70.8%)	302(67.0%)	120(82.8%)		173(76.5%)	113(70.6%)	60(90.9%)	
	**Follow-up 1**				**Follow-up 2**			
**Characteristics**	**Total**	**Responders**	**Non-responders**	***P***	**Total**	**Responders**	**Non-responders**	***P***
No.	235	176(74.9%)	59(25.1%)		135	115(85.2%)	20(14.8%)	
Age, year	45.74(23–72)	45.73(23–63)	46.31(24–72)	0.53	46.6(26–61)	46.87(26–61)	45.05(30–55)	0.34
FIGO stage								
IA2	1(0.4%)	0(0.0%)	1(1.7%)		1(0.7%)	1(1.7%)	0(0.0%)	
IB1	11(4.7%)	10(5.7%)	1(1.7%)		8(5.9%)	6(5.2%)	2(10.0%)	
IB2	50(21.3%)	41(23.3%)	9(15.3%)		21(15.6%)	19(16.5%)	2(10.0%)	
IIA	52(22.1%)	45(25.6%)	7(11.9%)		27(20.0%)	25(21.7%)	2(10.0%)	
IIB	94(40.0%)	68(38.6%)	26(44.%)		74(54.8%)	60(52.2%)	14(70.0%)	
IIIA	7(3.0%)	3(1.7%)	4(6.8%)		1(0.7%)	1(0.9%)	0(0.0%)	
IIIB	20(8.5%)	9(5.1%)	11(18.6%)		3(2.2%)	3(2.6%)	0(0.0%)	
IA2-IIA	114(48.5%)	96(54.5%)	18(30.5%)	<0.001	57(42.2%)	51(44.3%)	6(30.0%)	0.23
IIB-IIIB	121(51.5%)	80(45.5%)	41(69.5%)		78(57.8%)	64(55.7%)	14(70.0%)	
Histology								
Squamous cell carcinoma	208(88.5%)	154(87.5%)	54(91.5%)	0.40	135(100.0%)	115(100.0%)	20(100.0%)	
Adenocarcinoma^a^	27(11.5%)	22(12.5%)	5(8.5%)		0(0.0%)	0(0.0%)	0(0.0%)	
Tumor size								
<4	73(31.1%)	61(34.7%)	12(20.3%)	0.04	48(35.6%)	41(35.7%)	7(35.0%)	0.96
≥4	162(68.9%)	115(65.3%)	47(79.7%)		87(64.4%)	74(64.3%)	13(65.0%)	

^a^Adenocarcinoma and adenosquamous carcinoma were included.

**Table 2:** Summary results of four SNPs in the GWAS, case-control validation and the combined samples.

Chr	SNP	Position	Locus	Gene (±500 kb)	Stages	Sample size	aMA	*P* value	OR (95% CI)
4q34.3	rs6812281	180549803	intergenic	intergenic	Discovery	226	T	2.82E-05	2.64(1.68–4.15)
					Follow-up 1	235		7.90E-04	2.25(1.40–3.61)
					Follow-up 2	135		3.12E-02	2.09(1.07–4.09)
					Combined results^b^	461		9.04E-08	2.44(1.76–3.39)
					Combined results^c^	596		9.00E-09	2.37(1.77–3.18)
10q26.2	rs4590782	129550050	intergenic	BUB1P1, CLRN3, PTPRE, MKI67, LINC01163, DOCK1, NPS, FOXI2	Discovery	226	C	2.05E-05	0.29(0.17–0.51)
					Follow-up 1	235		2.48E-02	0.56(0.34–0.93)
					Follow-up 2	135		5.38E-01	0.79(0.37–1.69)
					Combined results^b^	461		6.67E-06	0.42(0.29–0.61)
					Combined results^c^	596		1.59E-05	0.48(0.34–0.67)
14q32.11	rs1742101	90234816	TTC7B	TTC7B, CALM1, NRDE2, PSMC1, RPS18P2, RPS18P2, RPS6KA5, RNU7-30P, C14orf159, SNORA11B	Discovery	226	A	4.29E-05	0.37(0.23–0.59)
					Follow-up 1	235		3.27E-02	0.63(0.42–0.96)
					Follow-up 2	135		1.58E-01	0.62(0.31–1.21)
					Combined results^b^	461		1.74E-05	0.50(0.37–0.69)
					Combined results^c^	596		7.11E-06	0.52(0.39–0.69)
16q23.3	rs1364121	82297608	CDH13	CDH13, MIR3182, HSBP1, MLYCO, OSGIN1, NECAB2, SLC38A8, RNA5SP432, MBTPS1, HSDL1, DNAAF1, TAF1C, ADAD2	Discovery	226	A	1.21E-04	2.49(1.56–3.96)
					Follow-up 1	235		3.16E-02	1.60(1.04–2.45)
					Follow-up 2	135		3.96E-02	2.09(1.04–4.22)
					Combined results^b^	461		2.85E-05	1.96(1.43–2.68)
					Combined results^c^	596		3.15E-06	1.98(1.49–2.64)

^a^MA: Minor allele; Combined results.

^b^Discovery and Follow-up 1 stages are combined; Combined results.

^c^Discovery, Follow-up 1, and Follow-up 2 stages are combined. OR: odds ratio for minor allele; 95% CI: 95% confidence intervals.
