# Supplementary material for: Genome-wide association study identifies four SNPs associated with response to platinum-based neoadjuvant chemotherapy for cervical cancer
Source: Sci Rep. 2017 Jan 25;7:41103. doi: 10.1038/srep41103 (PMC5264598; doi:10.1038/srep41103)
Supplement: Supplementary Information [file srep41103-s1.pdf]

Genome-wide association study identifies four SNPs associated with response to platinum-based neoadjuvant chemotherapy for cervical cancer

Xiong Li[1,2#], Kecheng Huang[1#], Qinghua Zhang[2#], Jin Zhou[1,3], Haiying Sun[1], Fangxu Tang[1], Hang Zhou[1], Ting Hu[1], Shaoshuai Wang[1], Yao Jia[1], Ru Yang[1], Yile Chen[4], Xiaodong Cheng[5], Weiguo Lv[5], Li Wu[4], Hui Xing[6], Lin Wang[1], Shasha Zhou[1], Yuan Yao[1], Xiaoli Wang[1], Quzhen Suolang[1], Jian Shen[2], Ling Xi[1], Junbo Hu[1], Hui Wang[1], Gang Chen[1], Qinglei Gao[1], Xing Xie[5], Shixuan Wang[1]*, Shuang Li[1]*, Ding Ma[1]*.

File list：

Figure S1. Quantile-Quantile plots of observed *P* values in –log10 scale of the discovery set.

Figure S2. Plots of the first two components derived from a principle component analysis of the discovery set implemented in the software package EIGENSTRAT.

Table S1
Table S2
Table S3
Table S4
Table S5
Table S6
Table S7
Table S8
Table S9

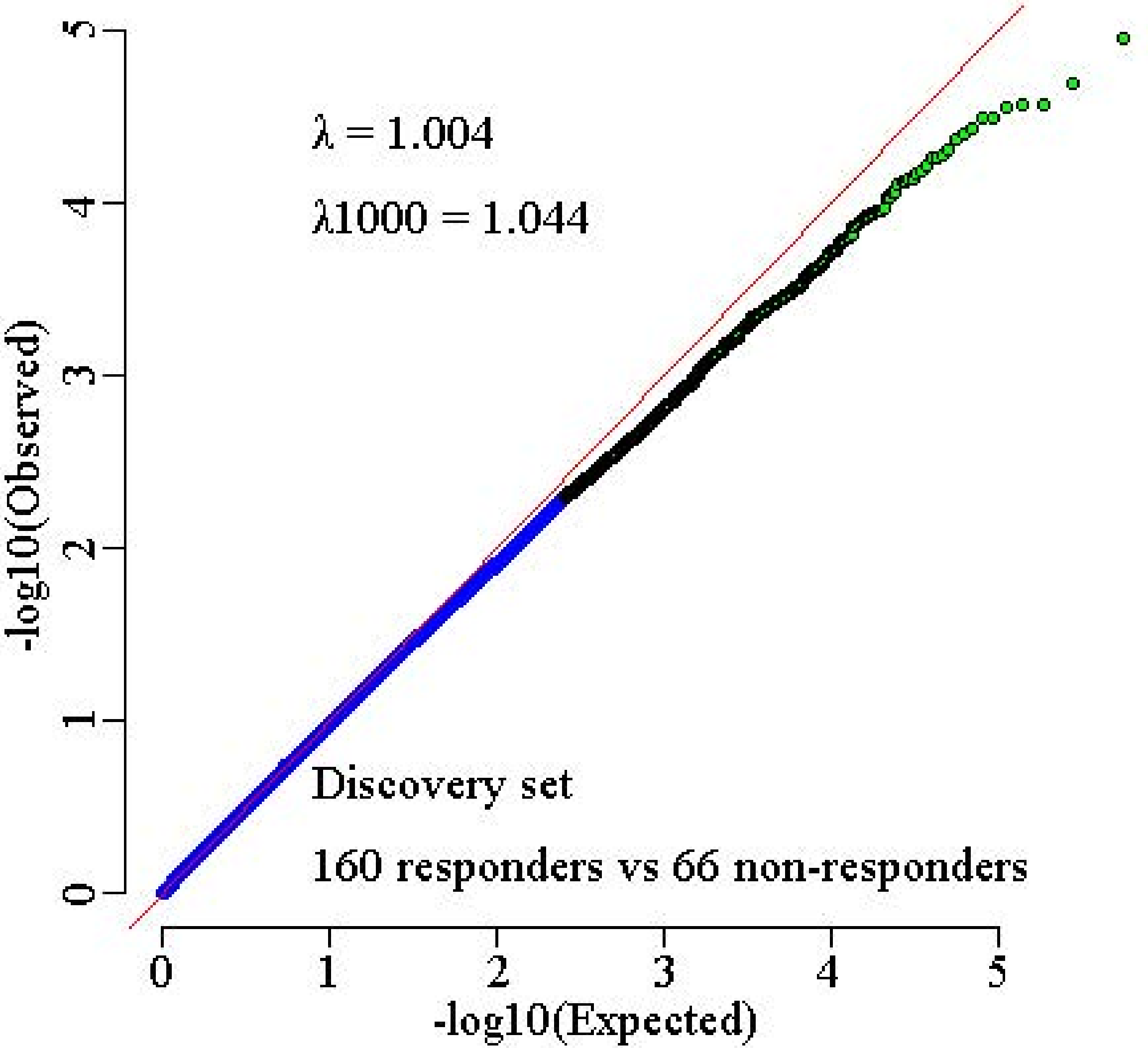

λ = 1.004
λ1000 = 1.044
Discovery set
160 responders vs 66 non-responders
-log10(Observed)
-log10(Expected)
0
1
2
3
4
5
0
1
2
3
4
5

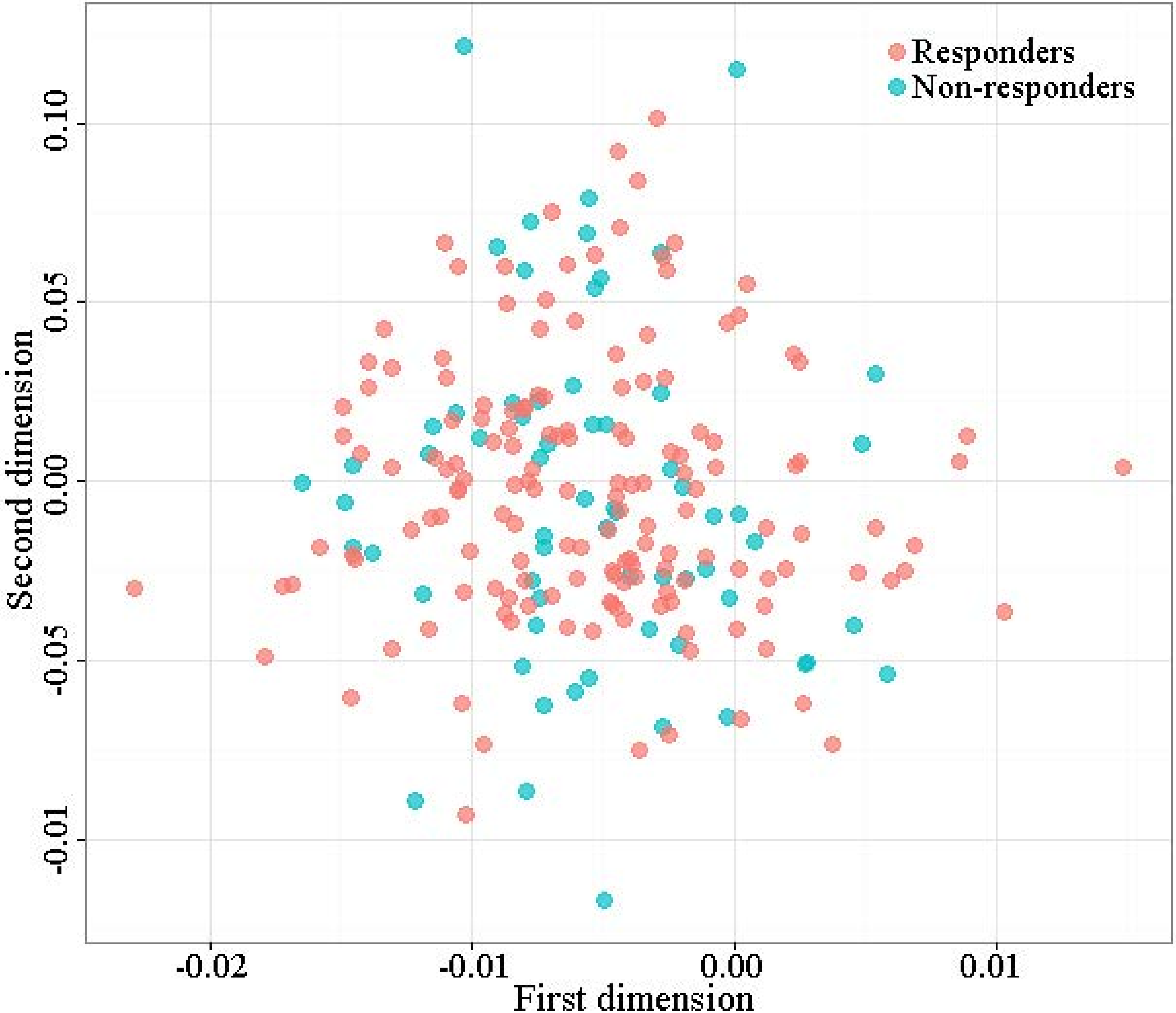
Responders
Non-responders
0.10
0.05
0.00
-0.05
-0.01
Second dimension
-0.02
-0.01
0.00
0.01
First dimension

Table S1. The geographical details of samples in the discovery stages and follow-up stages

		Discovery set		Follow-up 1		Follow-up 2	
	Total	CR+PR	SD+PD	CR+PR	SD+PD	CR+PR	SD+PD
Tong Ji	472	114（70.4%）	48（29.6%）	131（74.9%）	44（25.1%）	115（85.2%）	20（14.8%）
Xiang Yang	58	29（82.9%）	6（17.1%）	15（65.2%）	8（34.8%）	0	0
Zhe Jiang	66	17（58.6%）	12（41.4%）	30（81.1%）	7（18.9%）	0	0

CR, complete response; PR, partial response; SD, stable disease; PD, progressive disease.

Table S2. The top 100 ranked SNPs associated with response to NACT in discovery set

Rank	CHR	SNP code	SNP	Minor Allele	Major Allele	*P*
01	18	AX-11419637	rs28569227	A	G	1.11E-05
02	18	AX-11361410	rs2032256	A	G	1.12E-05
03	10	AX-11512751	rs4590782	C	T	2.05E-05
04	10	AX-12545784	rs2924264	C	T	2.65E-05
05	10	AX-16084295	rs10904352	A	G	2.65E-05
06	4	AX-11587201	rs6812281	T	G	2.82E-05
07	14	AX-12441597	rs12589908	C	T	3.17E-05
08	14	AX-12762082	rs7152201	C	T	3.24E-05
09	19	AX-13432569	NA	A	G	3.71E-05
10	2	AX-13940911	NA	C	T	3.95E-05
11	14	AX-11323533	rs1742101	A	G	4.29E-05
12	14	AX-11355992	rs1957867	A	G	4.87E-05
13	3	AX-11259897	rs1398920	A	G	5.27E-05
14	4	AX-14605488	rs72692490	T	C	5.39E-05
15	18	AX-12545268	rs2902932	A	G	5.51E-05
16	14	AX-11128513	rs10873453	C	T	6.00E-05
17	8	AX-11185666	rs11993811	T	C	6.50E-05
18	8	AX-15892712	rs4074052	A	G	6.69E-05
19	4	AX-14614851	rs7697454	C	T	7.13E-05
20	14	AX-11386819	rs2356907	G	A	7.27E-05
21	1	AX-12723359	rs1408951	G	C	7.37E-05
22	15	AX-12903004	rs1522771	G	C	7.59E-05
23	10	AX-11513811	rs4617528	G	A	7.86E-05
24	2	AX-12607692	rs6736735	A	G	8.48E-05
25	12	AX-12435476	rs12422261	C	T	8.62E-05
26	11	AX-16478049	rs73002702	A	C	9.24E-05
27	5	AX-15121462	rs61536869	A	G	9.34E-05
28	4	AX-11298016	rs17014118	T	C	1.07E-04
29	15	AX-11359918	rs2013555	C	T	1.09E-04
30	17	AX-12541147	rs28477485	A	G	1.11E-04
31	6	AX-11683163	rs9363346	G	T	1.12E-04
32	4	AX-14600493	rs62340307	G	A	1.14E-04
33	14	AX-12648031	rs8019419	T	C	1.15E-04
34	2	AX-14048336	NA	A	G	1.18E-04
35	5	AX-14855571	NA	A	G	1.19E-04
36	16	AX-12459820	rs1364121	A	G	1.21E-04
37	2	b36_2_1778789_f	rs10203567	C	T	1.23E-04
38	17	AX-12650042	rs8082149	T	C	1.27E-04
39	12	AX-16843218	rs2686344	T	C	1.30E-04
40	18	AX-13335901	rs756193	C	T	1.34E-04
41	10	AX-16407409	rs7897955	T	G	1.39E-04
42	6	AX-15278714	NA	T	G	1.39E-04

43	7	AX-11309212	rs17135914	C	T	1.54E-04
44	3	AX-12635128	rs7641032	T	G	1.55E-04
45	14	AX-12762348	rs36112191	A	G	1.56E-04
46	1	AX-11221715	rs1266382	T	C	1.57E-04
47	23	AX-12418198	rs1174067	T	C	1.62E-04
48	16	AX-11614432	rs7205784	T	C	1.63E-04
49	2	AX-13791339	rs10206154	G	A	1.72E-04
50	4	AX-11589257	rs6841508	A	G	1.72E-04
51	8	AX-15987054	rs10089846	C	T	1.74E-04
52	8	AX-11246337	rs13250856	A	G	1.81E-04
53	20	AX-12451106	rs13037957	C	T	1.85E-04
54	14	AX-11095721	rs10144133	T	G	1.85E-04
55	15	AX-11115636	rs10518980	A	G	1.89E-04
56	2	AX-13993043	NA	A	G	1.89E-04
57	15	AX-11106541	rs10431856	C	T	1.90E-04
58	21	AX-13648194	rs11088137	A	G	1.95E-04
59	23	AX-11171917	rs1174086	T	C	2.02E-04
60	14	AX-12527030	rs2362156	C	T	2.11E-04
61	3	AX-14115882	NA	A	G	2.12E-04
62	8	AX-11678893	rs9297524	C	T	2.15E-04
63	7	AX-11103765	rs10278689	T	C	2.22E-04
64	13	AX-11409742	rs2770526	G	A	2.25E-04
65	9	AX-16168232	NA	G	A	2.26E-04
66	17	AX-11114879	rs10515130	G	A	2.27E-04
67	11	AX-16478061	rs10502111	A	G	2.29E-04
68	8	AX-12412657	rs11365183	G	-	2.39E-04
69	3	AX-14208216	rs73039231	T	C	2.40E-04
70	10	AX-11504308	rs4405202	A	G	2.40E-04
71	20	AX-11556400	rs6038580	T	C	2.40E-04
72	20	AX-11556966	rs6048417	T	C	2.43E-04
73	14	AX-12826124	rs4903444	A	T	2.50E-04
74	7	AX-15692343	rs67747013	C	T	2.53E-04
75	3	AX-14208328	rs73041350	A	C	2.53E-04
76	2	AX-13993012	NA	A	G	2.56E-04
77	8	AX-16070538	rs6994772	A	G	2.57E-04
78	7	AX-15692353	rs58333121	C	T	2.60E-04
79	4	AX-11086761	rs10003747	A	G	2.64E-04
80	4	AX-11088293	rs10028840	C	G	2.67E-04
81	18	AX-12543341	rs28680308	T	C	2.75E-04
82	12	AX-16835759	NA	T	C	2.75E-04
83	11	AX-16472407	rs73547582	A	G	2.86E-04
84	20	AX-11560425	rs6127015	C	T	2.96E-04
85	10	AX-12652808	rs857919	T	G	2.98E-04
86	7	AX-11646681	rs7804000	T	C	3.02E-04

87	16	AX-12584940	rs4888769	T	C	3.04E-04
88	8	AX-11246864	rs13259384	A	G	3.04E-04
89	15	AX-12482258	rs16967544	G	A	3.07E-04
90	22	AX-13884964	rs7290423	A	G	3.08E-04
91	12	AX-11457316	rs35041751	A	G	3.10E-04
92	1	AX-11563361	rs6428430	T	C	3.12E-04
93	1	AX-16443160	rs4907947	T	G	3.13E-04
94	15	AX-13001181	rs6496033	A	C	3.17E-04
95	8	AX-16085368	rs12549682	A	G	3.17E-04
96	18	AX-11254922	rs1346970	G	A	3.28E-04
97	10	AX-11137783	rs11010917	C	A	3.30E-04
98	8	AX-11092576	rs10097592	G	A	3.30E-04
99	11	AX-16494089	NA	A	G	3.32E-04
100	3	AX-14208304	NA	T	G	3.33E-04

Table S3. Associations results of 23 SNPs in the discovery stage of GWAS.

Chr	SNP	Position	Associated Gene	A1	F_A	F_U	A2	*P* value	OR	95% CI
1p34.2	rs1408951	42178338	HIVEP3	C	0.33	0.15	G	7.37E-05	2.61	1.63-4.20
1q43	rs1266382	234748787	LGALS8	T	0.61	0.42	C	1.57E-04	2.36	1.51-3.69
2p25.3	rs10203567	1778789	MYT1L	G	0.54	0.33	A	1.23E-04	2.38	1.53-3.70
2q32.2	rs10206154	190680503	C2orf88	G	0.32	0.16	A	1.72E-04	2.64	1.59-4.38
3p24.3	rs1398920	18578309	SATB1-AS1	A	0.48	0.28	G	5.27E-05	2.70	1.67-4.38
3p24.3	rs7641032	23118465	intergenic	G	0.34	0.54	T	1.55E-04	0.43	0.28-0.66
4q34.3	rs6812281	180549803	RP11-404J23.1	T	0.39	0.18	G	2.82E-05	2.64	1.68-4.15
4q34.3	rs72692490	182375540	intergenic	T	0.31	0.13	C	5.39E-05	2.96	1.75-5.01
5p15.31	rs61536869	7926918	MTRR	A	0.26	0.11	G	9.34E-05	3.06	1.75-5.37
6q12	rs9363346	66106249	EYS	G	0.20	0.41	T	1.12E-04	0.38	0.23-0.62
8q24.23	rs4074052	139785008	COL22A1	T	0.13	0.32	C	6.69E-05	0.31	0.17-0.55
10p15.1	rs10904352	4885323	AKR1E2	A	0.39	0.19	G	2.65E-05	2.62	1.67-4.11
10q26.2	rs4590782	129550050	intergenic	C	0.14	0.35	T	2.05E-05	0.29	0.17-0.51
11q22.3	rs73002702	108539938	intergenic	A	0.37	0.18	C	9.24E-05	2.49	1.58-3.93
12q24.31	rs2686344	120174931	CAMKK2	T	0.42	0.23	C	1.30E-04	2.51	1.57-4.01
14q21.1	rs36112191	41583443	intergenic	A	0.27	0.45	G	1.56E-04	0.38	0.23-0.63
14q22.1	rs8019419	50551662	TRIM9	T	0.29	0.49	C	1.15E-04	0.41	0.26-0.64
14q32.11	rs1742101	90234816	TTC7B,RP11-61G16.2	A	0.29	0.50	G	4.29E-05	0.37	0.23-0.59
14q32.13	rs10873453	94085297	intergenic	C	0.28	0.11	T	6.00E-05	2.96	1.74-5.04
16q23.3	rs1364121	82297608	CDH13	A	0.43	0.25	G	1.21E-04	2.49	1.56-3.96
18p11.21	rs2902932	11086296	PIEZO2	A	0.36	0.18	G	5.51E-05	2.78	1.69-4.57
18q21.2	rs28569227	48778137	DCC	A	0.30	0.11	G	1.11E-05	3.40	1.97-5.86
Xq27.3	rs1174067	144345972	intergenic	A	0.41	0.23	G	1.62E-04	2.49	1.55-4.00

A1: Minor allele; F_A: Frequency of minor allele in cases; F_U: Frequency of minor allele in controls; A2: Major allele;

OR: odds ratio for minor allele; 95% CI: 95% confidence intervals.

Table S4. Associations results of 5 SNPs in the Follow-up 1 set.

Chr	SNP	Position	Associated Gene	A1	F_A	F_U	A2	*P* value	OR	95% CI
4	rs6812281	180549803	intergenic	T	0.37	0.21	G	7.90E-04	2.25	1.40-3.61
10	rs4590782	129550050	intergenic	C	0.22	0.33	T	2.48E-02	0.56	0.34-0.93
14	rs8019419	50551662	TRIM9	T	0.32	0.41	C	8.35E-02	0.68	0.44-1.05
14	rs1742101	90234816	TTC7B,RP11-61G16.2	A	0.38	0.50	G	3.27E-02	0.63	0.42-0.96
16	rs1364121	82297608	CDH13	A	0.42	0.31	G	3.16E-02	1.60	1.04-2.45

A1: Minor allele; F_A: Frequency of minor allele in cases; F_U: Frequency of minor allele in controls; A2: Major allele; OR: odds ratio for minor allele; 95% CI: 95% confidence intervals.

Table S5. Association results of 23 SNPs in the discovery study, two validations and the combined samples.

		Discovery study (160 CR+PR vs. 66 SD+PD)			Follow-up 1 (176CR+PR vs. 59 SD+PD)		
Chr	SNP	MA	*P* value	OR(95% CI)	MA	*P* value	OR(95% CI)
1	rs1408951	C	7.37E-05	2.61(1.63-4.20)	C	6.18E-01	1.12(0.73-1.71)
1	rs1266382	T	1.57E-04	2.36(1.51-3.69)	T	5.51E-01	0.87(0.56-1.37)
2	rs10203567	G	1.23E-04	2.38(1.53-3.70)	G	3.39E-01	0.80(0.50-1.27)
2	rs10206154	G	1.72E-04	2.64(1.59-4.38)	G	2.29E-01	0.71(0.40-1.24)
3	rs1398920	A	5.27E-05	2.70(1.67-4.38)	A	8.48E-01	0.96(0.61-1.49)
3	rs7641032	G	1.55E-04	0.43(0.28-0.66)	G	3.61E-01	0.83(0.55-1.24)
4	rs6812281	T	2.82E-05	2.64(1.68-4.15)	T	7.90E-04	2.25(1.40-3.61)
4	rs72692490	T	5.39E-05	2.96(1.75-5.01)	T	2.87E-01	0.73(0.41-1.31)
5	rs61536869	A	9.34E-05	3.06(1.75-5.37)	A	9.41E-01	1.02(0.61-1.70)
6	rs9363346	G	1.12E-04	0.38(0.23-0.62)	G	4.55E-01	1.18(0.77-1.81)
8	rs4074052	T	6.69E-05	0.31(0.17-0.55)	T	7.54E-01	1.08(0.68-1.69)
10	rs10904352	A	2.65E-05	2.62(1.67-4.11)	A	6.48E-01	0.89(0.53-1.49)
10	rs4590782	C	2.05E-05	0.29(0.17-0.51)	C	2.48E-02	0.56(0.34-0.93)
11	rs73002702	A	9.24E-05	2.49(1.58-3.93)	A	9.78E-01	0.99(0.60-1.64)
12	rs2686344	T	1.30E-04	2.51(1.57-4.01)	T	1.51E-01	1.39(0.89-2.17)
14	rs36112191	A	1.56E-04	0.38(0.23-0.63)	A	2.87E-01	0.78(0.49-1.24)
14	rs8019419	T	1.15E-04	0.41(0.26-0.64)	T	8.35E-02	0.68(0.44-1.05)
14	rs1742101	A	4.29E-05	0.37(0.23-0.59)	A	3.27E-02	0.63(0.42-0.96)
14	rs10873453	C	6.00E-05	2.96(1.74-5.04)	C	5.28E-01	0.82(0.44-1.52)
16	rs1364121	A	1.21E-04	2.49(1.56-3.96)	A	3.16E-02	1.60(1.04-2.45)
18	rs2902932	A	5.51E-05	2.78(1.69-4.57)	A	8.08E-01	1.06(0.65-1.73)
18	rs28569227	A	1.11E-05	3.40(1.97-5.86)	A	1.22E-01	1.49(0.90-2.46)
23	rs1174067	A	1.62E-04	2.49(1.55-4.00)	A	7.64E-01	1.08(0.67-1.72)

Table 5(continued)**.** Association results of 23 SNPs in the discovery study, two validations and the combined samples.

		Combined_Matched (336 CR+PR vs. 125 SD+PD)				
Chr	SNP	[a]MA	P_{fix} value	OR_{fix}(95% CI)	P_{ran} value	OR_{ran} (95% CI)
1	rs1408951	C				
1	rs1266382	T				
2	rs10203567	G				
2	rs10206154	G				
3	rs1398920	A				
3	rs7641032	G				
4	rs6812281	T	9.04E-08	2.44(1.76-3.39)	9.04E-08	2.44(1.76-3.39)
4	rs72692490	T				
5	rs61536869	A				
6	rs9363346	G				
8	rs4074052	T				
10	rs10904352	A				
10	rs4590782	C	6.67E-06	0.42(0.29-0.61)	6.73E-03	0.41(0.21-0.78)
11	rs73002702	A				
12	rs2686344	T				
14	rs36112191	A				
14	rs8019419	T	8.90E-05	0.53(0.39-0.73)	1.27E-02	0.53(0.32-0.87)
14	rs1742101	A	1.74E-05	0.50(0.37-0.69)	8.89E-03	0.49(0.29-0.84)
14	rs10873453	C				
16	rs1364121	A	2.85E-05	1.96(1.43-2.68)	2.04E-03	1.98(1.28-3.04)
18	rs2902932	A				
18	rs28569227	A				
23	rs1174067	A				

CR, complete response; PR, partial response; SD, stable disease; PD, progressive disease; [a]MA: Minor allele; OR: odds ratio for minor allele; 95% CI: 95% confidence intervals. P_{fix} value: P value for the fixed effect model; P_{ran} value: P value for the random effect model; OR_{fix}: Odds ratio for the fixed effect model; OR_{ran}: Odds ratio for the random effect model.

Table S6. Associations results of 5 SNPs in the Follow-up 2.

Chr	SNP	A1	F_A	F_U	A2	*P* value	OR	95% CI
4	rs6812281	T	0.40	0.22	G	3.12E-02	2.09	1.07-4.09
10	rs4590782	C	0.23	0.27	T	5.38E-01	0.79	0.37-1.69
14	rs8019419	T	0.33	0.37	C	5.76E-01	0.82	0.41-1.64
14	rs1742101	A	0.35	0.48	G	1.58E-01	0.62	0.31-1.21
16	rs1364121	A	0.45	0.28	G	3.96E-02	2.09	1.04-4.22

A1: Minor allele; F_A: Frequency of minor allele in cases; F_U: Frequency of minor allele in controls; A2: Major allele; OR: odds ratio for minor allele; 95% CI: 95% confidence intervals.

Table S7. Association results of 23 SNPs in the discovery study, two validations and the combined samples.

		Discovery study (160 CR+PR vs. 66 SD+PD)			Follow-up 1 (176CR+PR vs. 59 SD+PD)			Follow-up 2 (115 CR+PR vs. 20 SD+PD)		
Chr	SNP	[a]MA	*P* value	OR(95% CI)	[a]MA	*P* value	OR(95% CI)	[a]MA	*P* value	OR(95%CI)
1	rs1408951	C	7.37E-05	2.61(1.63-4.20)	C	6.18E-01	1.12(0.73-1.71)			
1	rs1266382	T	1.57E-04	2.36(1.51-3.69)	T	5.51E-01	0.87(0.56-1.37)			
2	rs10203567	G	1.23E-04	2.38(1.53-3.70)	G	3.39E-01	0.80(0.50-1.27)			
2	rs10206154	G	1.72E-04	2.64(1.59-4.38)	G	2.29E-01	0.71(0.40-1.24)			
3	rs1398920	A	5.27E-05	2.70(1.67-4.38)	A	8.48E-01	0.96(0.61-1.49)			
3	rs7641032	G	1.55E-04	0.43(0.28-0.66)	G	3.61E-01	0.83(0.55-1.24)			
4	rs6812281	T	2.82E-05	2.64(1.68-4.15)	T	7.90E-04	2.25(1.40-3.61)	T	3.12E-02	2.09(1.07-4.09)
4	rs72692490	T	5.39E-05	2.96(1.75-5.01)	T	2.87E-01	0.73(0.41-1.31)			
5	rs61536869	A	9.34E-05	3.06(1.75-5.37)	A	9.41E-01	1.02(0.61-1.70)			
6	rs9363346	G	1.12E-04	0.38(0.23-0.62)	G	4.55E-01	1.18(0.77-1.81)			
8	rs4074052	T	6.69E-05	0.31(0.17-0.55)	T	7.54E-01	1.08(0.68-1.69)			
10	rs10904352	A	2.65E-05	2.62(1.67-4.11)	A	6.48E-01	0.89(0.53-1.49)			
10	rs4590782	C	2.05E-05	0.29(0.17-0.51)	C	2.48E-02	0.56(0.34-0.93)	C	5.38E-01	0.79(0.37-1.69)
11	rs73002702	A	9.24E-05	2.49(1.58-3.93)	A	9.78E-01	0.99(0.60-1.64)			
12	rs2686344	T	1.30E-04	2.51(1.57-4.01)	T	1.51E-01	1.39(0.89-2.17)			
14	rs36112191	A	1.56E-04	0.38(0.23-0.63)	A	2.87E-01	0.78(0.49-1.24)			
14	rs8019419	T	1.15E-04	0.41(0.26-0.64)	T	8.35E-02	0.68(0.44-1.05)	T	5.76E-01	0.82(0.41-1.64)
14	rs1742101	A	4.29E-05	0.37(0.23-0.59)	A	3.27E-02	0.63(0.42-0.96)	A	1.58E-01	0.62(0.31-1.21)
14	rs10873453	C	6.00E-05	2.96(1.74-5.04)	C	5.28E-01	0.82(0.44-1.52)			
16	rs1364121	A	1.21E-04	2.49(1.56-3.96)	A	3.16E-02	1.60(1.04-2.45)	A	3.96E-02	2.09(1.04-4.22)
18	rs2902932	A	5.51E-05	2.78(1.69-4.57)	A	8.08E-01	1.06(0.65-1.73)			
18	rs28569227	A	1.11E-05	3.40(1.97-5.86)	A	1.22E-01	1.49(0.90-2.46)			
23	rs1174067	A	1.62E-04	2.49(1.55-4.00)	A	7.64E-01	1.08(0.67-1.72)			

Table S7.(continued)**.** Association results of 23 SNPs in the discovery study, two validations and the combined samples.

		Combined (451 CR+PR vs. 145 SD+PD)					
Chr	SNP	[a]MA	P_{fix} value	OR_{fix}(95% CI)	P_{ran} value	OR_{ran} (95% CI)	I^2
1	rs1408951						
1	rs1266382						
2	rs10203567						
2	rs10206154						
3	rs1398920						
3	rs7641032						
4	rs6812281	T	9.00E-09	2.37(1.77-3.18)	9.00E-09	2.37(1.77-3.18)	0.00
4	rs72692490						
5	rs61536869						
6	rs9363346						
8	rs4074052						
10	rs10904352						
10	rs4590782	C	1.59E-05	0.48(0.34-0.67)	1.02E-02	0.49(0.28-0.84)	59.49
11	rs73002702						
12	rs2686344						
14	rs36112191						
14	rs8019419	T	1.45E-04	0.57(0.43-0.76)	9.92E-03	0.59(0.39-0.88)	46.77
14	rs1742101	A	7.11E-06	0.52(0.39-0.69)	4.58E-04	0.52(0.36-0.75)	36.24
14	rs10873453						
16	rs1364121	A	3.15E-06	1.98(1.49-2.64)	3.15E-06	1.98(1.49-2.64)	0.00
18	rs2902932						
18	rs28569227						
23	rs1174067						

CR, complete response; PR, partial response; SD, stable disease; PD, progressive disease; [a]MA: Minor allele; OR: odds ratio for minor allele; 95% CI: 95% confidence intervals. P_{fix} value: P value for the fixed effect model; P_{ran} value: P value for the random effect model; OR_{fix}: Odds ratio for the fixed effect model; OR_{ran}: Odds ratio for the random effect model; I^2: Heterogeneity index of the meta-analysis of three stages.

Table S8. Association between SNP genotypes and clinicopathologic factors.

	FIGO stage			Histology			Turmor size		
SNP	IA2-IIA	IIB-IIIB	*P*	Squamous cell carcinoma	Adenocarcinoma[a]	*P*	<4	≥4	*P*
rs6812281									
GG	169	176	0.36	318	27	0.51	106	239	0.30
GT+TT	132	118		234	16		67	183	
rs4590782									
TT	155	146	0.68	280	21	0.82	86	215	0.74
CT+CC	147	148		273	22		88	207	
rs1742101									
GG	81	89	0.36	154	16	0.19	42	128	0.12
AG+AA	220	205		398	27		132	293	
rs1364121									
GG	132	146	0.16	260	18	0.51	79	199	0.68
GA+AA	169	148		292	25		95	222	

[a] Adenocarcinoma and adenosquamous carcinoma were included

Table S9. Logistic analysis of SNP genotypes with response to neoadjuvant chemotherapy.

	Univariate analysis			Multivariate analysis		
	CR+PR	SD+PD	*P*	Hazard ratio	95%CI	*P*
rs6812281						
GG	288	57		1		
GT+TT	162	88	1.621E-7	2.621	1.755-3.914	2.47E-06
rs4590782						
TT	207	94		1		
CT+CC	244	51	7.312E-5	0.472	0.314-0.710	3.04E-04
rs1742101						
GG	116	54		1		
GA+AA	334	91	7..875E-3	0.693	0.453-1.060	NS
rs1364121						
GG	228	50		1		
GA+AA	222	95	6.816E-4	1.937	1.286-2.916	1.55E-03
Tumor size						
<4	149	25		1		
≥4	302	120	2.734E-4	2.381	1.452-3.904	5.86E-04

CR, complete response; PR, partial response; SD, stable disease; PD, progressive disease; 95% CI: 95% confidence intervals.
